# An extremely sensitive nested PCR-RFLP mitochondrial marker for detection and identification of salmonids in eDNA from water samples

**DOI:** 10.7717/peerj.3045

**Published:** 2017-02-28

**Authors:** Laura Clusa, Alba Ardura, Sara Fernández, Agustín A. Roca, Eva García-Vázquez

**Affiliations:** 1 Department of Functional Biology, University of Oviedo, Oviedo, Asturias, Spain; 2 USR3278-CRIOBE-CNRS-EPHE-UPVD, Laboratoire d’Excellence “CORAIL”, Université de Perpignan, Perpignan, France

**Keywords:** eDNA, Species-specific RFLP, Family-specific primers, Salmonids

## Abstract

**Background:**

Salmonids are native from the North Hemisphere but have been introduced for aquaculture and sport fishing in the South Hemisphere and inhabit most rivers and lakes in temperate and cold regions worldwide. Five species are included in the Global Invasive Species Database: rainbow trout *Oncorhynchus mykiss*, Atlantic salmon *Salmo salar*, brown trout *Salmo trutta*, brook trout *Salvelinus fontinalis*, and lake trout *Salvelinus namaycush*. In contrast, other salmonids are endangered in their native settings.

**Methods:**

Here we have developed a method to identify salmonid species directly from water samples, focusing on the Iberian Peninsula as a case study. We have designed nested Salmonidae-specific primers within the 16S rDNA region. From these primers and a PCR-RFLP procedure the target species can be unequivocally identified from DNA extracted from water samples.

**Results:**

The method was validated in aquarium experiments and in the field with water from watersheds with known salmonid populations. Finally, the method was applied to obtain a global view of the Salmonidae community in Nalón River (north coast of Spain).

**Discussion:**

This new powerful, very sensitive (identifying the species down to 10 pg DNA/ml water) and economical tool can be applied for monitoring the presence of salmonids in a variety of situations, from checking upstream colonization after removal of river barriers to monitoring potential escapes from fish farms.

## Introduction

Salmonids are a fish group particularly interesting because, although native from the north Hemisphere, they are spread worldwide. Many species have been introduced into streams for recreational fishing (Hasegawa & Maekawa, 2006) and for aquaculture. Five species: *Oncorhynchus mykiss*, *Salmo salar*, *Salmo trutta*, *Salvelinus fontinalis* and *Salvelinus namaycush* are considered invasive and included in the Global Invasive Species Database (GISD) (http://www.issg.org/database) of the IUCN. Moreover, brown trout (*S. trutta*) and rainbow trout (*O. mykiss*) are within the One Hundred of the World’s Worst Invasive Alien Species list, which includes species that cause serious negative impacts on biological diversity and/or human activities (Lowe et al., 2000).

The five salmonids listed above have been introduced worldwide. From its native range in Europe and North Africa, brown trout (*S. trutta*) has expanded to all continents except Antarctica (MacCrimmon & Marshall, 1968). Rainbow trout (*O. mykiss*) was one of the most widely exported salmonids in the beginning of the 20th century, for aquaculture from the USA to 28 out of 41 European countries (Crawford & Muir, 2008; Savini et al., 2010; Stanković, Crivelli & Snoj, 2015) including Spain (Elvira & Almodóvar, 2001). Atlantic salmon (*S. salar*), one of the most consumed fish in the world, can be found out of its North Atlantic native area as far as in Australia, New Zealand, Chile, West Coast of the US and Canada (De Poorter, 2009). Lake trout (*S. namaycush*) was also introduced in Europe from North America and Canada for recreational fishing. Some populations were established in deep, high-altitude lakes in the French Pyrenees and in alpine lakes in Switzerland (Crossman, 1995). Brook trout (*S. fontinalis*) is also established in some European countries: France, Austria, Germany, Switzerland etc. (MacCrimmon & Campbell, 1969). In Spain it can be found in Tagus and Ebro rivers and Cantabrian lakes (Doadrio, 2001).

The use of these exotic species for aquaculture and their accidental and intentional release or escape negatively impacts on native biodiversity and ecosystems (Hewitt, Campbell & Gollasch, 2006). To cite just a few examples, introduced salmonids have endangered native biota to the extinction or near-extinction of vulnerable species in Australia (Morgan et al., 2004), New Zealand (Townsend, 1996; Townsend, 2003), Argentina (Consuegra et al., 2011), Japan (Kitano, 2004) or Canada (Dextrase & Mandrak, 2006). Brook trout strongly impacted on the endemic Iberian frog *Rana iberica* (Bosch et al., 2006). Rainbow trout outcompeted other salmonid species for space and food (Stanković, Crivelli & Snoj, 2015), as brown trout (Levin et al., 2002) and brook trout (Nakano et al., 1998; Blanchet et al., 2007) also did in different regions of North America and Europe.

On the other hand, many salmonid populations are endangered in their native settings. A paradigmatic example is the decline of Atlantic salmon (*S. salar)* in their native rivers over the northern Hemisphere (Klemetsen et al., 2003; Jelks et al., 2008; Hórreo et al., 2011; Chaput, 2012). In Europe, 41% of native Salmonidae species are threatened (Freyhof & Brooks, 2011). The Atlantic salmon has undergone historical extirpation from rivers in Belgium, Czech Republic, Germany, Netherlands, Poland, Slovakia and Switzerland (Freyhof, 2014). Atlantic salmon populations from Duero, Tagus and Guadiana rivers are now extinct in Spain (Baillie & Groombridge, 1996), where only a few rivers in the north still support wild populations that are in continuous decline (Hórreo et al., 2011); their status is considered vulnerable (Freyhof, 2014). The reasons for this are principally habitat losses (damming, pollution), overfishing, and the introduction of invasive species (Chown et al., 2015). Infections, probably coming from fish farms, have also threatened European Atlantic salmon (Krkošek et al., 2007; Whelan, 2010), as well as other species such as the Arctic char (*Salvelinus alpinus*) and sea trout (*S. trutta*) in Norway (Bjørn, Finstad & Kristoffersen, 2001). During the 20th century, wild populations of brown trout decreased in Finland due to dams construction and overfishing (Syrjänen & Valkeajärvi, 2010). In other countries, like in Spain, some local sedentary populations of *S. trutta* (Doadrio, 2001) have been totally extirpated. This species is considered vulnerable in Spain (Freyhof, 2011).

In the last years, molecular tools such as barcoding and metabarcoding are becoming very useful for managing natural populations and communities (Chown et al., 2015). An emerging method to monitor and detect aquatic species is environmental DNA (eDNA) analysis. Metazoans can be detected from their DNA released into the environment through skin flaking and sloughed cells, mucus excretion and defecation in aquatic environments (Goldberg, Strickler & Pilliod, 2015). In many cases, eDNA amplification from PCR seems to be more sensitive and efficient than traditional surveillance approaches, like visual detection, and does not disturb the aquatic fauna (Ficetola et al., 2008; Blanchet, 2012; Thomsen et al., 2012). The use of specific primers on eDNA has been successfully demonstrated for a number of species. Examples are molluscs such as *Rangia cuneata* in the Baltic Sea (Ardura et al., 2015), *Xenostrobus securis* and *Potamopyrgus sp* in North Spain (Devloo-Delva et al., 2016; Clusa et al., 2016); fishes such as *Petromyzon marinus* (Gustavson et al., 2015), *Neogobius melanostomus* (Adrian-Kalchhauser & Burkhardt-Holm, 2016), *Cyprinus carpio* (Uchii, Doi & Minamoto, 2016). Salmonids with designed specific methodology based on eDNA include, amongst others, *Salvelinus namaycush* (Lacoursière-Roussel et al. , 2015), *Salmo trutta* (Gustavson et al., 2015; Carim et al., 2016), *Oncorhynchus mykiss* (Wilcox et al., 2015), *Salvelinus fontinalis* (Wilcox et al., 2013). These methods can be applied to detect, and in some cases roughly quantify, elusive or threatening species even at very low density. Methodologies based on eDNA may be particularly useful for inventorying salmonids from running or turbid waters, when traditional electrofishing or netting methods are not efficient (for example in reservoirs), and indeed when those sampling methods may disturb other vulnerable species cohabiting the same watersheds.

In this work, we developed a method based on PCR-RFLP (Polymerase chain reaction-restriction fragment length polymorphism) mitochondrial marker to detect the presence of salmonid species from water samples, in order to monitoring the presence of salmonid species focusing on North Iberia as a model region study. This region is interesting because it contains two native Salmoninae, *S. salar* and *S. trutta*, and three exotic species considered invasive by the International Union of Conservation of Nature (rainbow, brook and lake trout) that were introduced in rivers and lakes decades ago (Doadrio, 2001; Elvira & Almodóvar, 2001; Crawford & Muir, 2008). This was the second eDNA method validated for identifying Salmonidae from European water samples, after the *S. trutta* specific primer described by Gustavson et al. (2015), and the first method to detect salmonid mixtures from a single PCR. The mixture of introduced and native species makes North Iberia a good case study for application of eDNA methodology to monitoring of feral populations. PCR-RFLP methodology has been successful for identification of different fish species (e.g., Itoi et al., 2005; Reid & Wilson, 2006), but has been employed on community DNA from aquatic samples only on protozoans (Xiao et al., 2000; Galván et al., 2014). Since it is relatively inexpensive, technically easy and fast, if successful from eDNA, it could be widely applied in ecology, conservation biology and management of aquatic resources in many zones of Europe, especially in the Atlantic Arc where the aquatic fauna is similar to North Spain’s.

## Materials and Methods

### Salmonidae specific primers

To obtain enough PCR product to perform the RFLP analysis, a nested PCR strategy was used. The method here described was based on the DNA fragment amplified with Salmonidae-specific primers described in Zaiko et al. (2015). We designed a new primer pair to nest Zaiko et al. (2015) primers inside its amplification product. The 16S rRNA gene was chosen because, as a mitochondrial gene, it is more abundant than nuclear DNA in water samples (Ficetola et al., 2008), it is generally well conserved within species and exhibits higher variation between species (Maretto et al., 2007; Zhang & Hanner, 2012). All the 16S rRNA gene sequences available for the Salmonidae species *O. mykiss*, *S. trutta, S. salar, S. namaycush* and *S. fontinalis* were downloaded from the NCBI database of DNA sequences, either individual 16S DNA sequences or complete mitochondrial genomes. Polymorphisms were analyzed with the DNASP software V.5.10 (Rozas et al., 2003). The different haplotypes were visualized employing the BioEdit Sequence Alignment Editor software (Hall, 1999). Sequences were aligned with the ClustalW application included in BioEdit (Thompson, Higgins & Gibson, 1994). The Primer Blast application included in the NCBI webpage (Ye et al., 2012) was employed to design one forward 16S general primer, which amplified a fragment of 567 bp in the 16S rRNA gene using the reverse 16S-Br universal primer from Palumbi et al. (2002).

The two Salmonidae-specific primers described by Zaiko et al. (2015) anneal within the 567 bp amplicon obtained from the new primers pair. These Salmonidae specific primers were tested *in silico* with the BLAST tool in the NCBI webpage (Altschul et al., 1990). The sequences retrieved with significant match (e-value of 0.046 for the forward primer and 0.18 for the reverse) were from the Order Salmoniformes. Both forward and reverse primers were checked, and the two BLAST results were contrasted to determine which species will probably amplify with both of them. One non-target species with significant match *in silico* was *Esox lucius*, which is an invasive species in the region and was included in the RFLP designed, to avoid any false positive after digestion.

To validate the new Salmonidae-specific primer *in vitro*, cross-amplification tests were performed. Samples from different species belonging to 15 fish families from the laboratory collection were used. They represent the 100% of the species inventoried in the study area, north coast of Spain (Table S1). DNA was extracted from muscle tissue with Chelex resine (Estoup et al., 1996). PCR amplifications were performed with the universal primers for the 16S gene (Palumbi et al., 2002), sequenced to confirm the species and used as DNA quality control. The primers pair was tested for PCR amplification on all the samples of Table S1 and *O. mykiss, S. salar, S. trutta, S. fontinalis* and *S. namaycush* as positive controls. The PCR conditions were as described in ‘PCR conditions’ but using 2 µl of template DNA extracted from tissue samples. We assayed the following annealing temperatures: 58°C, 60°C, 62°C, 64°C, 66°C and 68°C; and the following MgCl_2_ concentrations: 2.5 mM, 2 mM, 1.5 mM, 1 mM. The best conditions were selected.

### PCR-RFLP method development

The RFLP (restriction fragment length polymorphism) protocol was designed within the DNA fragment amplified with the primers described by Zaiko et al. (2015). To design species-specific RFLPs, all the haplotypes from the five species in study were aligned. Diagnostic single nucleotide sites (monomorphic within species and different between species) were identified for each species. The restriction enzymes recognizing those sites were selected and restriction pattern determined using the NEBcutter application (Vincze, Posfai & Roberts, 2003). For the species *S. namaycush* there were not enough sequences in the database. DNA extracted from 25 samples of this species kindly provided by the Université Laval of Québec were amplified with the universal primers for the 16S RNA gene (Palumbi et al., 2002) and sequenced. The new sequences were employed for RFLP design.

### PCR conditions

In the first PCR, a fragment of 567 bp in the 16S rRNA gene was amplified with the forward 16S general new primer (see ‘Salmonidae specific primers’) and the reverse 16S-Br universal primer from Palumbi et al. (2002). The amplification reaction was performed in a total volume of 20 µl, including Green GoTaq^®^Buffer 1X, 2.5 mM MgCl_2_, 0.25 mM dNTPS, 1 µM of each primer, 4 µl of template DNA, 200 ng/µl of BSA (bovine serum albumin) and 0.65 U of DNA Taq polymerase (Promega). PCR conditions were the same as described by Palumbi et al. (2002), but with 50 cycles instead of 35. Both negative control with only distilled water and positive control with *S. salar* DNA from tissue were included. This PCR confirmed the quality of DNA in the sample and discard false negatives due to excessive DNA degradation, and was used as template for the nested-PCR, amplifying a smaller fragment of the 16S rRNA gene with the Salmonidae specific primers.

The nested PCR amplification with the pair of Salmonidae-specific primers described in Zaiko et al. (2015) was performed in a total volume of 20 µl, including Green GoTaq^®^Buffer 1X, MgCl_2_, 0.25 mM dNTPS, 1 µM of each primer, 200 ng/µl of BSA and 0.5 µl of PCR product from the previous 16S amplification as template and 0.65 U of DNA Taq polymerase (Promega). The PCR conditions were the following: an initial denaturation step at 95°C for 5 min, 35 cycles at 94°C for 30 s, annealing at the temperature of choice for 30 s and elongation at 72°C for 30 s. A final step of elongation was set at 72°C for 10 min. In nested PCR two negative and two positive controls were included, one negative with only distilled water and another negative using as template the PCR product from the negative control in the first PCR and the same with the positive controls. PCR products were visualized in 2% agarose gels with 2.5 µl of SimplySafe™.

### Restriction enzyme digestion validation

The PCR product amplified with the nested PCR described above was digested with FastDigest enzymes (Thermo Scientific). The digestion reaction was performed in a total volume of 15 µl, including 5 µl of PCR product (approximately 100 ng of DNA), 1.5 µl of Green Buffer 10X, 0.3 µl of Enzyme and 8.2 µl H_2_O. The incubation time was 10 min at 37°C for the *HindIII*, *SchI* and *VspI* enzymes and 10 min at 65°C for *TaaI* and *Tru1I*. The five species were digested with all the enzymes in order to validate the restriction pattern. The PCR-RFLP method was assayed using mixtures with different proportion of *Salvelinus namaycush* and *S. fontinalis* DNA as templates.

### Sensitivity of the method

The detection limit of direct PCR with the two Salmonidae-specific primers alone (without nested PCR) was determined from serial dilutions of DNA of the five species (*S. salar, S.trutta, O. mykiss, S. fontinalis and S. namaycush*), starting from a known concentration (1 µg/ml).

The detection limit of the nested PCR (16S PCR followed of a PCR from the amplification fragment with the two Salmonidae-specific primers as described in ‘PCR conditions’) was done also from the same serial dilutions employed above. The dilution where no amplification was observed in agarose gel was considered the detection limit. DNA concentration was measured with a fluorometer Qubit^®^dsDNA BR Assay.

To test the sensitivity of the PCR and RFLP, several mixes of *S. namaycush* were tested. Mix 1 with 37.5 ng of DNA from *S. namaycush* and 12.5 ng of *S. fontinalis*, Mix 2 with 25 ng of each species, Mix 3 with 12.5 ng of *S. namaycush* and 37.5 ng of *S. fontinalis*, Mix 4 with 5 ng of *S. namaycush* and 45 of *S. fontinalis* and Mix 5 with 50 ng DNA of 6 Salmonidae species (*S. namaycush, S. fontinalis, S. alpinus, S. trutta, S. salar and O. mykiss*).

### Method validation for eDNA

The method was validated in environmental DNA from aquarium samples as well as from field water samples obtained in locations with known salmonid populations.

Aquarium tests included two experimental situations: one of high density with six *Salmo trutta* juveniles (mean weight 1.714 ± 0.301 g) and another of low density with three *S. trutta* juveniles (two replicates: mean weights of 1.537 ± 0.405 g for Replica 1 and 1.400 ± 0.865 g for Replica 2). The brown trout juveniles were left swimming in aquariums of 15 L for 5 days. Everyday 10 L of water were replaced, after five days one sample of 1 L of water was taken from each aquarium for filtration and DNA extraction (see below) and nested PCR-RFLP was done.

**Figure 1:**
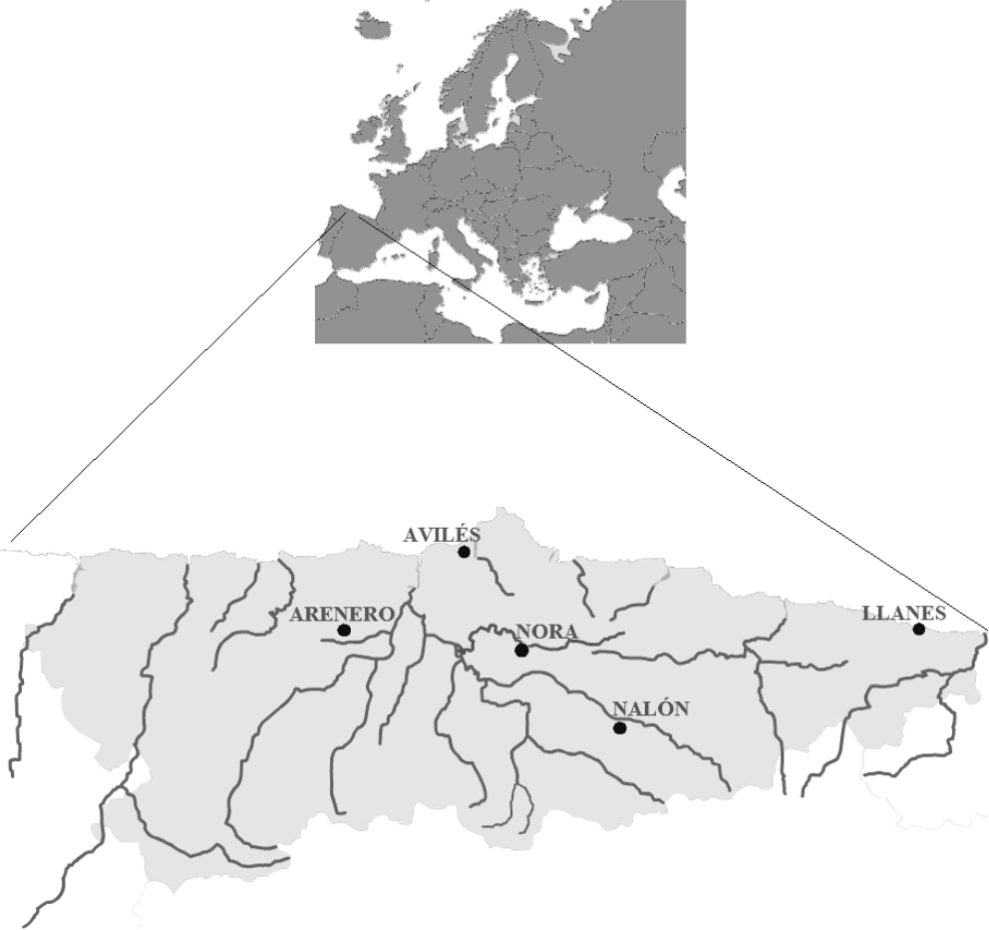
Map from Asturias (Spain). Water sampling sites: River Nora, River Nalón, fishing reservoir “El Arenero”, Avilés and Llanes are shown.

For validation with field samples, one liter of water was collected from two positive and two negative control sites in the region of Asturias (north of Spain; Fig. 1). One positive control was Nora River (Asturias, north of Spain) at the coordinates 43.379283N, −5.788667W, with an average discharge of 20.98 m^3^/s. This tributary of Nalón River is isolated from the mainstream due to an impassable dam and contains a small resident population of *Salmo trutta*. No other salmonids or fish farms occur in the river. The other positive sample was the fishing reservoir “El Arenero” at the coordinates 43.346814N, −6.378065W. This one hectare surface pond contains *O. mykiss* released by the managers. The negative controls were the estuary of Aviles (coordinates 43.573223N, −5.922922W) and the Llanes Beach (coordinates 43.420461N, −4.752003W), where there are no salmonids.

Filtration and DNA extraction was done as explained in ‘Measures for avoiding contamination in eDNA.’ Nested PCR and RFLP were applied on the DNA extracted from the water samples as described in ‘PCR conditions’ and ‘Restriction enzyme digestion validation.’ All samples were tested with the five restriction enzymes.

### Measures for avoiding contamination in eDNA

Two separate areas were used for the whole process, one for pre-PCR and another one for post-PCR. Filtration of water samples was done in the pre-PCR room, where there were no positive DNA or tissue samples. Water samples were vacuum filtered using the Supor^®^-200 Membrane Filter (Pall Corporation) with 0.2 µm pore size and a reusable filter holder. The filter holder was dismantled, sprayed with 10% bleach, cleaned with detergent and 10% bleach, rinsed with distilled water and autoclaved between each sampling site. To ensure the cleaning process was correct, one sample with 1 L distilled water was filtrated between two problem samples and included in all eDNA analyses to confirm that contamination did not occur in the filtration or extraction process.

DNA was extracted with the PowerWater^®^ DNA Isolation Kit (QIAGEN laboratories). The eDNA extraction was done in a separate laboratory unit inside a PCR laminar flow cabinet treated with ultraviolet light, where no salmonid tissue sample has never been used. The process was done using filter tips, to avoid contamination of the extraction kit and between samples.

The PCR reaction was prepared in the pre-PCR room inside a PCR cabinet treated with ultraviolet light. Once every sample was ready, closed and inside the PCR machine, the positive control was added in the post-PCR room and put into the machine, to avoid any contact between tubes with samples and with positive control. In every step, negative controls were added to ensure the samples were contamination free, as explained above.

### Case study: Nalón River

**Figure 2:**
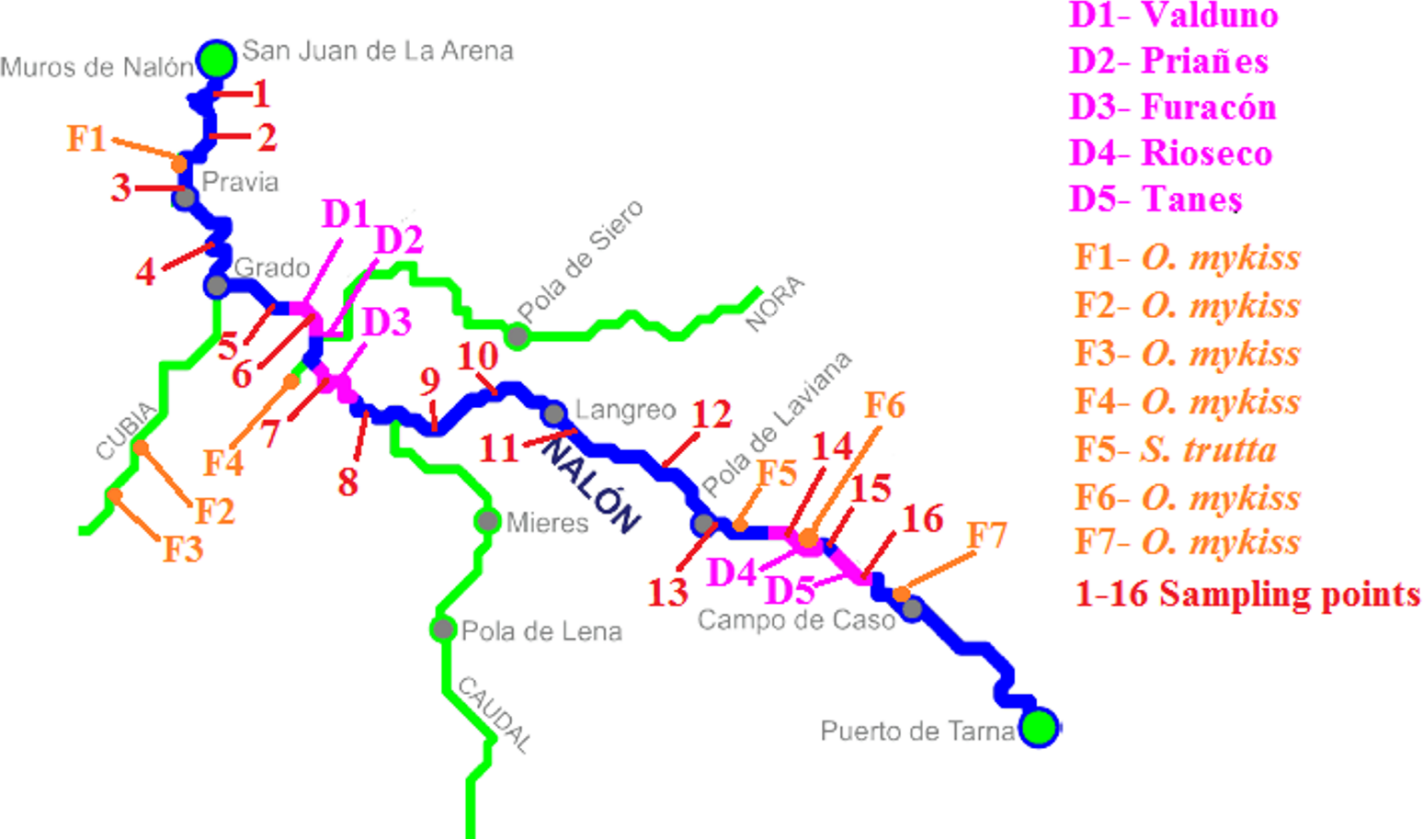
Nalón River basin. Dams along the river are shown; from downstream to upstream they are Valduno (D1), Priañes (D2), Furacón (D3), Rioseco (D4) and Tanes (D5). The fish farms are pointed as F1 to F7 and finally the sampling points are numbered in red from 1 to 16.

The method was applied in Nalón River (Cantabrian corridor basin), of 140.8 km long and with an average discharge of 55.18 m^3^/s. There are five dams in its way long: Valduno, Priañes, Furacón, Rioseco and Tanes from downstream to upstream (D1–D5 respectively in Fig. 2). Seven fish farms are located along the river: one downstream in Pravia (Piscifactoría Barganeiro) where *O. mykiss* is farmed (F1); two in Cubia River, a tributary of Nalón River (Piscifactoría Alcubiella and Piscifactoría del Alba III), both with *O. mykiss* (F2 and F3); one in Somines (Piscifactoría Somines) with *O. mykiss* (F4); one in Laviana (Piscifactoría La Chalana) where *S. trutta* is reared (F5); and two upstream (Piscifactoría del Alba SA I in Soto de Agues and Piscifactoría del Nalón I in Veneros) with *O. mykiss* (F6, F7) (Ministerio de Agricultura, Alimentación y Medio Ambiente, 2016).

In February 2016, 16 different points along Nalón River were selected and sampled (1–16 in Fig. 2). Three liters of water were collected with sterile bottles from each point from upstream to downstream, putting the bottle as close to the bottom substrate as possible. They were put in ice and transported rapidly to the laboratory. Two samples of 1 L were filtered and extracted as described above, and one liter was stored frozen for confirmatory analysis if needed. Each replicate was extracted and analyzed separately in time. With the two eDNA samples, the PCR-RFLP method was performed twice, to discard false positive and false negative results due to technical failure. A minimum of two positive results from different extractions were considered valid to corroborate the presence of a species in the sample. In some cases where only one of the two eDNA replicates were positive, the PCR-RFLP method was performed three times to consider the result as positive. When the digestion results were not clear (too weak bands), the digestion was repeated using 10 µl of PCR template instead of 5 µl as described above. All the samples were tested with all the restriction enzymes.

### Ethics statement

This project and the experimental procedure including aquarium stage of *Salmo trutta* was approved by the Committee of Ethics of the Government of the Principality of Asturias according to the Royal Decree 53/2013 of 1 February 2013 that regulates the use of experimental animals in Spain, with the permit code PROAE 25/2015.

## Results

### Primers designed

The new general forward primer was: 16S-new-F (5′-GCCTGCCCTGTGACTATGG-3′). Together with the universal 16S-Br reverse primer (Palumbi et al., 2002), they amplify a fragment of 567 nucleotides within the 16S rRNA gene, located between the sites 2046 and 2613 of the *Salmo salar* mitochondrion complete genome (GenBank: KF792729.1). The Salmonidae specific primers designed *in silico* from the analysis of databases and new sequences of salmonids were:

Forward primer: 16S-F-Salm (5′-AAGACCTGTATGAATGGCATC-3′)

Reverse primer: 16S-R-Salm (5′-TCGATAGGGACTCTGGGAGA-3′).

These primers amplify a fragment of 377 nucleotides within the 16S rRNA gene, located between the sites 2125 and 2502 of the same *S. salar* reference sequence used before. The assays of annealing temperatures for the PCR with Salmonidae specific primers showed that the best results were obtained at 68°C with 2 mM MgCl_2_. All the 16S rDNA sequences obtained and employed in this work are available in GenBank with the accession numbers stated in Table S1. In cross-amplification assays we have confirmed that the new primers only amplified from salmonids species. The sequence of the amplicons obtained for *O. mykiss*, *S. trutta*, *S. salar*, *S. fontinalis* and *S. namaycush* are available in GenBank with the accession numbers KU510521, KU510522, KU510523, KU510525 and KU510526 respectively.

The threshold of detection for direct PCR with the two Salmonidae-specific primers- and visualization in agarose gels was 0.1 ng/ml. We observed a band of the expected size in the dilution 1 to 10,000 from the five tested samples with an initial concentration of 1 µg/ml.

The detection limit for the nested PCR method (16S PCR with the new general primer designed and one universal Palumbi’s primer followed by a PCR with the two Salmonidae-specific primers) was 10 pg/ml, since positive bands of the expected amplicon size were observed in agarose from the dilution 1 to 100,000 of the five samples with an initial concentration of 1 µg/ml.

### The species-specific PCR-RFLP

**Figure 3:**
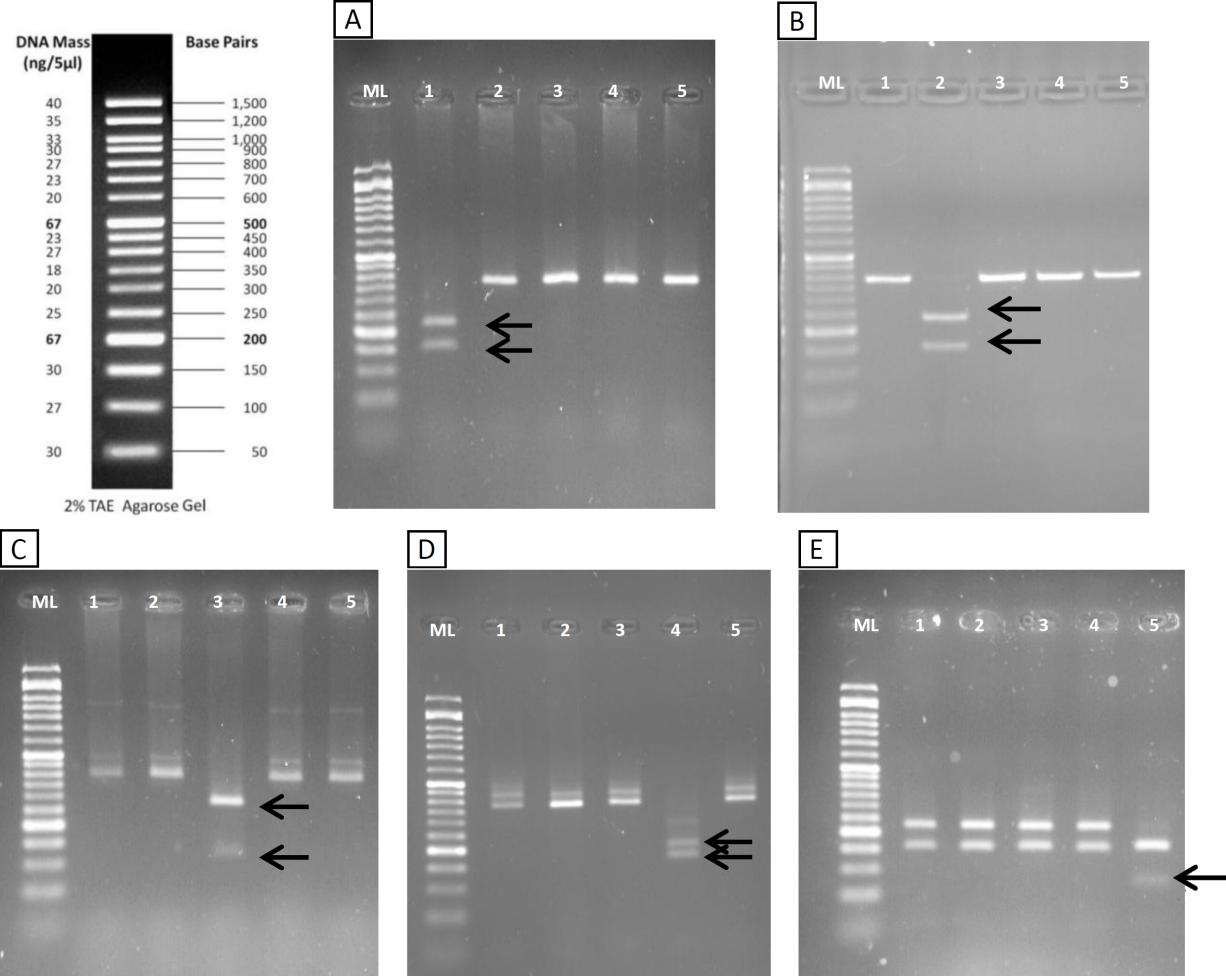
RFLP validation. Agarose gels (2%) showing results of digestion with VspI (A), HindIII (B), SchI (C), TaaI (D) and Tru1I (E). Lanes (from 1 to 5) in all gels are: Ladder (ML), *S. fontinalis*, *S. namaycush*, *S. salar*, *S. trutta*, *O. mykiss*. Diagnostic bands for each species are marked with arrows.

The restriction patterns within the fragment amplified with the new primers provided specific bands for all the considered species (Table 1). The diagnostic bands could be clearly differentiated in agarose gel (Fig. 3). Specific bands for *S. fontinalis* were 222 bp and 155 bp with the enzyme VspI, specific bands for *S. namaycush* 231 bp and 146 bp with HindIII, and so on. The non-target species *Esox lucius*, which has significant match with the primers, theoretically amplifies a fragment of 373 nt and was included in the design of the RFLP. None of the enzymes are supposed to digest the amplicon, except Tru1I which could give fragments of 140, 138, 75 and 21 bp. These fragments are clearly different from the diagnostic bands of the target species. Thus the RFLP patterns were well defined and allowed to differentiating each species.

**Table 1:** Restriction patterns obtained with the enzymes considered for the five salmonid species. The bands in bold are diagnostic to identify each species.

Enzyme	FastDigest	Restriction site	Species	Bands	Rest of species
*HindIII*	FD0504	AAGCTT	*Salvelinus namaycush*	**231** and **146** bp	377 bp
*VspI*	FD0914	ATTAAT	*Salvelinus fontinalis*	**222** and **155** bp	377 bp
*SchI*	FD1374	GAGTC(N)_5_	*Salmo salar*	**272** and **103** bp	374 y 3 bp
*TaaI*	FD1364	ACNGT	*Salmo trutta*	**205** and **172** bp	377 bp
*Tru1I*	FD0984	TTAA	*Oncorhynchus mykiss*	155, 156 and **66** bp	222, 150 and 5 bp

Regarding the sensitivity of this method in agarose gels, in mixes of *S. namaycush* (lake trout) and *S. fontinalis* (brook trout) DNA (Table S2) it was possible to observe the clear diagnostic band of *S. namaycush* down to as little as 5ng of *S. namaycush* DNA. Moreover a light band could also be observed in Mix 5, where the DNA template was a mixture of 5 different species with only 7.5% of *S. namaycush* DNA. Thus this method was effective for recognizing a species also when there were different Salmonid species mixed in a site.

### Detection of salmonids from water samples

**Figure 4:**
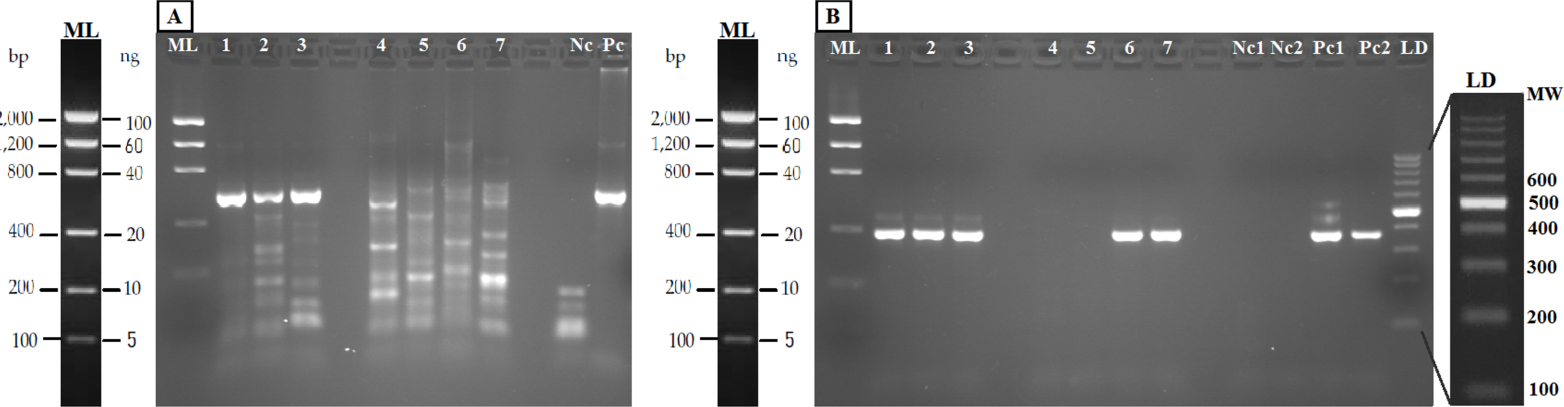
Agarose gels (2%) showing validation of the method with eDNA. Samples in both geles are aquarium samples: BThd (1), BT1 (2), BT2 (3), and field samples: Llanes (4), Avilés (5), Nora River (6) and fishing reservoir “El Arenero” (7), negative and positive controls are included (Nc and Pc respectively). (A) PCR product from 16S general PCR and (B) PCR product from Nested PCR with Salmonidae specific primers. Positive controls (Pc1) and (Pc2): nested and direct PCR, respectively, on *Salmo salar* DNA extracted from muscle. Negative controls are indicated as (Nc1 and Nc2) nested and direct PCR, respectively.

The PCR-RFLP method was validated in both aquarium experiments and field water samples. The PCR was performed with the new general forward primer (16S-new-F) and the universal 16S-Br reverse primer (Palumbi et al., 2002). All the eDNA samples yielded amplification products of the expected size (Fig. 4A). Nested PCR provided a clear band of 370 nucleotides, the expected amplicon size, in both aquarium and positive field samples (Nora River and “El Arenero”); in the two other field samples no positive amplification was obtained from these primers, as expected since salmonids do not occur in Aviles estuary and Llanes beach (Fig. 4B).

**Figure 5:**
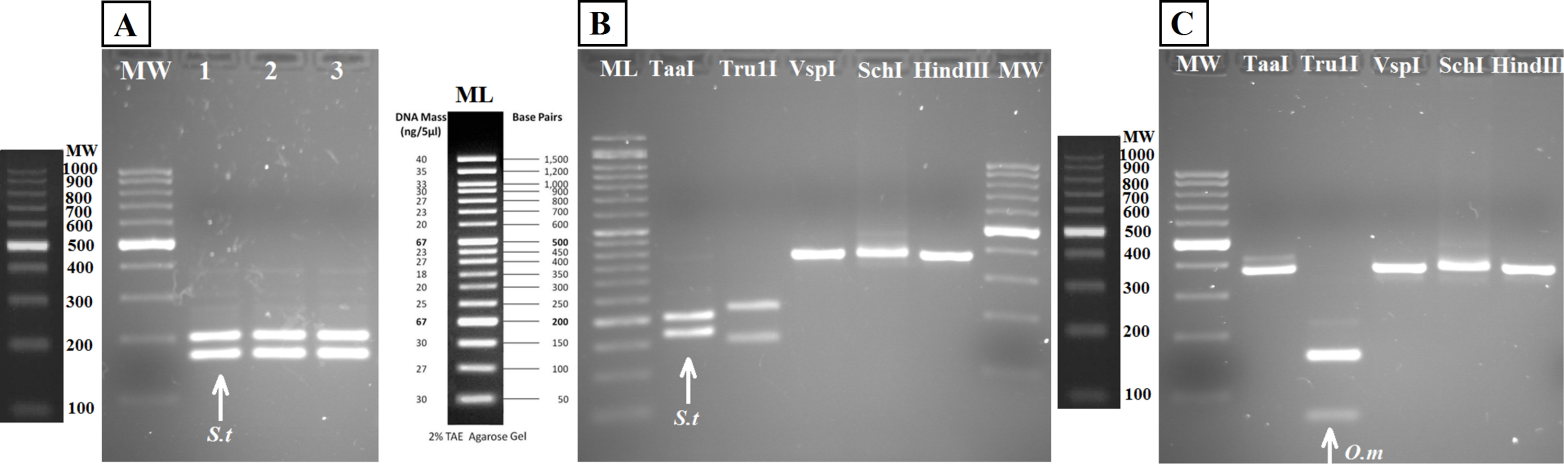
Agarose gels showing restriction fragments obtained after digestion of amplicons from water DNA samples (Fig. 4B). (A) Aquarium samples digested with TaaI, BThd (1), BT1 (2) and BT2 (3). (B) Restriction fragments of amplicons from nested PCR of Nora River water obtained with: TaaI, Tru1I, VspI, SchI, HindIII. (C) Restriction fragments of amplicons from nested PCR of “El Arenero” water, obtained with: TaaI, Tru1I, VspI, SchI, HindIII. Diagnostic bands of different species are marked with arrows. *Om* and *St* are *O. mykiss* and *S. trutta* respectively.

The bands typical of S. *trutta* (205 and 172 bp) were obtained after digestion with the enzyme *TaaI* (Fig. 5) in aquarium samples, thus validating the use of this PCR-RFLP marker from water eDNA.

The water sample from Nora River, with a known population of *S. trutta*, and the water sample from “El Arenero,” with a known population of *O. mykiss*, provided a clear band of 370 nucleotides with the Salmonid-specific primers (Fig. 4B). The PCR products obtained from River Nora and “El Arenero” water DNA were purified, sequenced and the sequence identified by BLAST as *S. trutta* and *O. mykiss* respectively. Both sequences are available in GenBank (Accession numbers KU510527 and KX904362).

RFLP digestions confirmed the species present in water samples in all cases (Figs. 5B and 5C). Typical bands of S. *trutta* (205 and 172 bp) were obtained after digestion with the enzyme *TaaI* in Nora River sample. In the sample from the fishing reservoir “El Arenero,” it is possible to identify *O. mykiss*; the bands expected of *O. mykiss* (66 bp) were obtained after digestion with *Tru1I*. This validates the method from field environmental samples with complex species mixtures, in both type of samples watercourse (Nora River) and ponds (“El Arenero”).

### Case study: Nalón River

The results of Nalón River are shown in Table 2. The sampling points were separated from each other by an average distance of 6.74 ± 3.02 km. Multiple Salmonidae species were detected from the same sample and with the same PCR product. *Salmo salar* was found downstream in points 1 to 3. It should be noted that Narcea River, which is a tributary of Nalón River and a well known Atlantic salmon preserve, joins the mainstream in Pravia (upstream point 3).

**Table 2:** Naló River results. The positive identification of the species in each point is showed with an “X” and the negative identification with “–”.

Sampling points	Coordinates	Distance between 1 point and the next (km)	*S. trutta*	*O. mykiss*	*S. salar*	*S. fontinalis*	*S. namaycush*
Sampling point 1 La Arena	43.548512N, −6.080661W	1.68	X	X	X	–	–
Sampling point 2 Soto del Barco	43.535637N, −6.080841W	10.49	X	X	X	–	–
Sampling point 3 Pravia	43.491283N, −6.103837W	7.90	X	X	X	–	–
Sampling point 4 San Román	43.448498N, −6.079927W	11.6	X	X	–	–	–
Sampling point 5 Bar Casa Aurina	43.403614N, −6.040419W	3.95	X	X	–	–	–
Sampling point 6 Valduno’s dam	43.38987N, −6.0053W	8.76	X	X	–	–	–
Sampling point 7 Trubia	43.354393N, −5.963959W	6.39	X	–	–	–	–
Sampling point 8 Las Caldas	43.331509N, −5.930557W	8.50	X	X	–	–	–
Sampling point 9 Soto de Ribera	43.308495N, −5.870101W	6.99	–	X	–	–	–
Sampling point 10 Olloniego	43.315437N, −5.814595W	11.85	–	–	–	–	–
Sampling point 11 Lada	43.306736N, −5.697167W	9.87	X	X	–	–	–
Sampling point 12 San Martín del Rey Aurelio	43.273834N, −5.601774W	6.65	X	X	–	–	–
Sampling point 13 Laviana	43.237621N, −5.554755W	9.14	X	X	–	–	–
Sampling point 14 Rioseco’s dam	43.223583N, −5.459807W	2.72	X	X	–	–	–
Sampling point 15 Anzó	43.225563N, −5.438601W	6.58	X	–	–	–	–
Sampling point 16 Tanes’ dam	43.192474N, −5.382222W	–	X	X	–	–	–

*S. trutta* and *O. mykiss* were found along the whole river. In points 7 and 15 only *S. trutta* eDNA was found, while in point 9 *O. mykiss* was the only species found from eDNA. In point 10 none of these salmonids were detected with the new marker.

## Discussion

Here we described a robust marker for detection and identification of five species of salmonids from water samples based on RFLP from the product of a single PCR. Specific PCR primers are available for different Salmonidae such as *S. namaycush* (Lacoursière-Roussel et al. , 2015), *S. trutta* (Gustavson et al., 2015; Carim et al., 2016), *O. mykiss* (Wilcox et al., 2015), *S. fontinalis* (Wilcox et al., 2013), but for European waters only one has been recently described for detection of brown trout (Gustavson et al., 2015). Our method enabled applications in a wider range of situations and species mixtures.

All previously described studies were based on qPCR, useful for knowing the density of one species. Our tool allows for a rapid overview of the Salmonidae community without the use of real-time PCR systems, and in the particular case of Spain it allowed to detect exotic and native salmonids at the same time. As it is, the method is ready to be used in Spanish waters, but it could be easily adapted for application in other region by checking for any cross-amplification with the local aquatic fauna.

Another advantage of the method was its technical accessible procedure that may allow to be routinely implemented in a laboratory, since it requires less special technical know-how or equipment than qPCR or NGS. RFLP-based methods are generally more economical and faster in comparison to metabarcoding (Teletchea, 2009; Li et al., 2015), and could be applied in routine sampling in a near future. The average cost of the method employed here was 13.4 euros per water sample including reagents for DNA extraction, PCR amplification and digestions with the complete set of enzymes (not the labor that may vary very much depending on salary wages and possible robotizing). The whole process would not take longer than one day, and it is possible to analyze several samples at the same time. It could also be robotized for genotyping in capillary electrophoresis using labeled primers, expectedly with better results because capillary electrophoresis has a better resolution than agarose gels. Compared with NGS metabarcoding, the digestion products can be directly interpreted and do not need bioinformatics analysis as NGS does (Coissac, Riaz & Puillandre, 2012; Taberlet & Coissac, 2012). DNA metabarcoding may be also limited by the difficulty to design universal primers (Deagle et al., 2014). Compared with other methodologies, such as SNPs (Wenne et al., 2016), it does not require high DNA quality; in fact in environmental samples the DNA is degraded and fragmented and despite it, it was possible to apply the method here described directly on water samples.

Since the Salmonidae-specific primers are highly sensitive, it is possible to use them directly on water samples for detecting salmonids, without the need of nested-PCR. This has been already proven from ballast water samples for confirming the presence of salmonid DNA detected from Next-Generation Sequencing (NGS) metabarcoding (Zaiko et al., 2015). Given its extreme sensitivity when using nested-PCR, our method could be applied in running waters. The method as it is could be applied to monitor the use of streams by Atlantic salmon, since it served to detect this species downstream the studied river. It could be used for a quick search of non-native populations of trout (e.g., Atlantic hatchery bred *S. trutta*) along classical DNA isolation from fin-clips. It could be easily adapted to coho salmon (*O. kisutch*) and sockeye salmon (*O. tshawytscha*) in Canada (Irvine et al., 2005), and other populations of Pacific salmon in the USA (Gustafson et al., 2007). It would be especially useful in protected spaces, enabling to detecting the presence of salmonids without disturbing wild populations with electrofishing.

On the other hand, it could be a useful tool to detect salmonids in places where these species are exotic and represent a danger to the local fauna. It could serve to detect escapes from aquaculture, a big problem for local wild populations (Hewitt, Campbell & Gollasch, 2006; De Poorter, 2009), and to detect populations of exotic salmonids—such as rainbow trout in Spain (Elvira & Almodóvar, 2001), or brown trout in New Zealand (Townsend, 1996; Townsend, 2003). In our case study (Nalón River) *S. trutta* and *O. mykiss* were detected from almost every sampling point, which we expected since there are *S. trutta* populations in Nalón River and some fish farms for *O. mykis.*

The biggest weakness of our method may be the mitochondrial sequences employed. Exotic salmonids can hybridize with native salmonids, since in this family genetically close species hybridize with each other (e.g., Rubidge & Taylor, 2004; Hórreo et al., 2011). Indeed, the marker here developed cannot detect hybrids because mitochondrial DNA has maternal inheritance. On the other hand it is based on DNA, a resistant molecule that can be amplified from dead animals, or from farm discharges. Hänfling et al. (2016) showed that eDNA from flowing streams may contaminate lake samples. Deiner & Altermatt (2014) demonstrated that eDNA from two invertebrates (*Daphnia longispina* and *Unio tumidus*) could be detected as far as nine to 12 km downstream from their populations were known to occur. Another contamination source could be avian feces, such as Merkes et al. (2014) showed. They found that the DNA of silver carp from avian excrement could be detected and the detection persisted for 28 days. Other authors have measured the degradation of eDNA in a ecosystem, such as Strickler, Fremier & Goldberg (2015), who tested the effect of UV, temperature and pH in *Lithobates catesbeianus* eDNA obtaining positive detection from one to 54 days after species removal. On the other hand, De Souza et al. (2016) suggested that eDNA detection probability for the two species *Necturus alabamensis* and *Sternotherus depressus* was strongly affected by the season of sampling. In our particular case study of Nalón River *O. mykiss* was not detected from point 15 (Table 2 and Fig. 2) which is 10.85 km downstream the closest fish farm (Piscifactoría Nalón I in Veneros, F7 in Fig. 2), but was identified from point 9 which is 47.08 km downstream the closest farm (Piscifactoría del Alba SA I in Soto de Agues, F6 in Fig. 2). We could interpret that, at least in the second case, the presence of O. *mykiss* DNA was probably due to real individuals, coming from escapes of fish farms. To confirm positive results in the wild when a contamination source of eDNA is near, such as fish farms, it would be advisable to survey the place at different times, and to confirm the presence of individual escapes from conventional physical sampling.

## Conclusion

This PCR-RFLP method is a sensitive tool able to detect the presence of five salmonid species by analyzing DNA extracted from water samples from a nested PCR and further simultaneous restriction digestions. This innovation may have various applications worldwide, either for detecting exotic salmonids or for monitoring native populations without disturbing them and the rest of aquatic fauna.

##  Supplemental Information

10.7717/peerj.3045/supp-1 Table S1 Samples from different families of fishes from the laboratory collection employed for testing the Salmonidae-specific markers All the 16S rDNA haplotypes from each species obtained and employed in this work are available in GenBank with the accession numbers stated in the table.

10.7717/peerj.3045/supp-2 Table S2 Sensitivity assays Mix 1–5: Mixes of *S. namaycush* and *S. fontinalis* DNA used in the development of the PCR-RFLP method, indicating the percentage of *S. namaycush* and the amount of DNA of each species in the mix. In bold are the diagnostic fragments that can be seen in the agarose gel.

## Additional Information and Declarations

### Data Availability

The following information was supplied regarding data availability: Genbank KU510485–KU510527 and KX904362
Clusa, Laura; Ardura, Alba; Fernández, Sara; Roca, Agustín; García-Vázquez, Eva (2017): An extremely sensitive nested PCR-RFLP mitochondrial marker for detection and identification of salmonids in eDNA from water samples. figshare.
https://doi.org/10.6084/m9.figshare.4597888.v1.
