# Supplementary material for: An extremely sensitive nested PCR-RFLP mitochondrial marker for detection and identification of salmonids in eDNA from water samples
Source: PeerJ. 2017 Feb 28;5:e3045. doi: 10.7717/peerj.3045 (PMC5333537; doi:10.7717/peerj.3045)
Supplement: Table S1 — All the 16S rDNA haplotypes from each species obtained and employed in this work are available in GenBank with the accession numbers stated in the table. [file peerj-05-3045-s001.docx]

| **Phylum** | **Class** | **Order** | **Family** | **Species** | **Accession numbers** |
| --- | --- | --- | --- | --- | --- |
| Chordata | Actinopterygii | Clupeiformes | Clupeidae | *Sardina pilchardus* | KU510505 |
| Chordata | Actinopterygii | Clupeiformes | Engraulidae | *Engraulis encrasicolus* | KU510488 |
| Chordata | Actinopterygii | Gadiformes | Gadidae | *Micromesistius poutassou* | KU510496 |
| Chordata | Actinopterygii | Gadiformes | Gadidae | *Pollachius pollachius* | KU510500 |
| Chordata | Actinopterygii | Gadiformes | Merlucciidae | *Merluccius merluccius* | KU510493-KU510494 |
| Chordata | Actinopterygii | Lophiformes | Lophiidae | *Lophius budegassa* | KU510492 |
| Chordata | Actinopterygii | Perciformes | Carangidae | *Trachurus* | KU510504 |
| Chordata | Actinopterygii | Perciformes | Gobiidae | *Gobius* | KU510489 |
| Chordata | Actinopterygii | Perciformes | Moronidae | *Dicentrarchus labrax* | KU510486 |
| Chordata | Actinopterygii | Perciformes | Mullidae | *Mullus surmuletus* | KU510497 |
| Chordata | Actinopterygii | Perciformes | Scombridae | *Scomber scombrus* | KU510503 |
| Chordata | Actinopterygii | Perciformes | Sparidae | *Boops boops* | KU510485 |
| Chordata | Actinopterygii | Perciformes | Sparidae | *Diplodus vulgaris* | KU510487 |
| Chordata | Actinopterygii | Pleuronectiformes | Pleuronectidae | *Platichthys flesus* | KU510498-KU510499 |
| Chordata | Actinopterygii | Pleuronectiformes | Scophthalmidae | *Lepidorhombus boscii* | KU510490 |
| Chordata | Actinopterygii | Pleuronectiformes | Scophthalmidae | *Lepidorhombus whiffiagonis* | KU510491 |
| Chordata | Actinopterygii | Pleuronectiformes | Scophthalmidae | *Psetta maxima* | KU510501 |
| Chordata | Actinopterygii | Pleuronectiformes | Scophthalmidae | *Scophthalmus rhombus* | KU510502 |
| Chordata | Actinopterygii | Pleuronectiformes | Soleidae | *Microchirus variegatus* | KU510495 |
| Chordata | Actinopterygii | Salmoniformes | Salmonidae | *Oncorhynchus mykiss* | KU510508 |
| Chordata | Actinopterygii | Salmoniformes | Salmonidae | *Salmo salar* | KU510514-KU510516 |
| Chordata | Actinopterygii | Salmoniformes | Salmonidae | *Salmo trutta* | KU510509-KU510513 |
| Chordata | Actinopterygii | Salmoniformes | Salmonidae | *Salvelinus alpinus* | KU510517 |
| Chordata | Actinopterygii | Salmoniformes | Salmonidae | *Salvelinus fontinalis* | KU510518 |
| Chordata | Actinopterygii | Salmoniformes | Salmonidae | *Salvelinus namaycush* | KU510519-KU510520 |
| Chordata | Actinopterygii | Zeiformes | Zeidae | *Zeus faber* | KU510506-KU510507 |
