# Supplementary material for: An extremely sensitive nested PCR-RFLP mitochondrial marker for detection and identification of salmonids in eDNA from water samples
Source: PeerJ. 2017 Feb 28;5:e3045. doi: 10.7717/peerj.3045 (PMC5333537; doi:10.7717/peerj.3045)
Supplement: Table S2 — Mix 1–5: Mixes of S. namaycush and S. fontinalis DNA used in the development of the PCR-RFLP method, indicating the percentage of S. namaycush and the amount of DNA of each species in the mix. In bold are the diagnostic fragments that can be seen in the agarose gel. [file peerj-05-3045-s002.docx]

| **Nº** | **Enzymes** | **Incubation** | **Mixes** | **% *S. namaycush*** | **Expected Bands** | **Detection** |
| --- | --- | --- | --- | --- | --- | --- |
| Mix 1 | HindIII | 37°C-10’ | *Salvelinus namaycush* 37.5 ng + *Salvelinus fontinalis* 12.5 ng | 75 | 377**, 231** and **146** bp | Positive |
| Mix 2 | HindIII | 37°C-10’ | *Salvelinus namaycush* 25ng + *Salvelinus fontinalis* 25 ng | 50 | 377**, 231** and **146** bp | Positive |
| Mix 3 | HindIII | 37°C-10’ | *Salvelinus namaycush* 12.5 ng + *Salvelinus fontinalis* 37.5 ng | 25 | 377**, 231** and **146** bp | Positive |
| Mix 4 | HindIII | 37°C-10’ | *Salvelinus namaycush* 5 ng + *Salvelinus fontinalis* 45 ng | 10 | 377**, 231** and **146** bp | Positive |
| Mix 5 | HindIII | 37°C-10’ | 2 µl each: *Salvelinus namaycush, Salvelinus fontinalis, Salvelinus alpinus, Salmo trutta, Salmo salar and Oncorhynchus mykiss.* | 7.15% (50 ng/700 ng DNA) | 377**, 231** and **146** bp | Positive |
